# Contribution of Molecular Structure to Self-Assembling and Biological Properties of Bifunctional Lipid-Like 4-(*N*-Alkylpyridinium)-1,4-Dihydropyridines

**DOI:** 10.3390/pharmaceutics11030115

**Published:** 2019-03-12

**Authors:** Martins Rucins, Pavels Dimitrijevs, Klavs Pajuste, Oksana Petrichenko, Ludmila Jackevica, Anita Gulbe, Signe Kibilda, Krisjanis Smits, Mara Plotniece, Dace Tirzite, Karlis Pajuste, Arkadij Sobolev, Janis Liepins, Ilona Domracheva, Aiva Plotniece

**Affiliations:** 1 Latvian Institute of Organic Synthesis, Aizkraukles str. 21, Riga LV-1006, Latvia; rucins@osi.lv (M.R.); p.dimitrijevs@osi.lv (P.D.); klavs.pajuste@osi.lv (K.P.); oksana.petricenko@lu.lv (O.P.); ludmila@farm.osi.lv (L.J.); anita.gulbe@farm.osi.lv (A.G.); tirzite@latnet.lv (D.T.); kpajuste@osi.lv (K.P.); arkady@osi.lv (A.S.); ilona@farm.osi.lv (I.D.); 2 Department of Pharmaceutical Chemistry, Faculty of Pharmacy, Riga Stradiņš University, Dzirciema str. 16, Riga LV-1007, Latvia; mara.plotniece@rsu.lv; 3 Faculty of Medicine, University of Latvia, Jelgavas str. 1, Riga LV-1004, Latvia; 4 Laboratory of Magnetic Soft Materials, Department of Physics, University of Latvia, Jelgavas str. 3, Riga LV-1004, Latvia; 5 JSC Grindex, Krustpils str 53, Riga LV-1057, Latvia; signe.kibilda@grindeks.lv; 6 Institute of Solid State Physics, University of Latvia, Kengaraga str. 8, Riga LV-1063, Latvia; smits@cfi.lu.lv; 7 Institute of Microbiology and Biotechnology, University of Latvia, Jelgavas str. 1, Riga LV-1004, Latvia; janis.liepins@lu.lv

**Keywords:** synthetic lipids, pyridinium and propargyl moieties, nanoparticles, self-assembling properties, DLS, TEM, cytotoxicity, toxicity on microorganisms, phospholipid binding

## Abstract

The design of nanoparticle delivery materials possessing biological activities is an attractive strategy for the development of various therapies. In this study, 11 cationic amphiphilic 4-(*N*-alkylpyridinium)-1,4-dihydropyridine (1,4-DHP) derivatives differing in alkyl chain length and propargyl moiety/ties number and position were selected for the study of their self-assembling properties, evaluation of their cytotoxicity in vitro and toxicity on microorganisms, and the characterisation of their interaction with phospholipids. These lipid-like 1,4-DHPs have been earlier proposed as promising nanocarriers for DNA delivery. We have revealed that the mean diameter of freshly prepared nanoparticles varied from 58 to 513 nm, depending upon the 4-(*N*-alkylpyridinium)-1,4-DHP structure. Additionally, we have confirmed that only nanoparticles formed by 4-(*N*-dodecylpyridinium)-1,4-DHP derivatives **3** and **6,** and by 4-(*N*-hexadecylpyridinium)-1,4-DHP derivatives **10** and **11** were stable after two weeks of storage. The nanoparticles of these compounds were found to be homogenous in size distribution, ranging from 124 to 221 nm. The polydispersity index (PDI) values of 1,4-DHPs samples **3**, **6**, **10**, and **11** were in the range of 0.10 to 0.37. We also demonstrated that the nanoparticles formed by 4-(*N*-dodecylpyridinium)-1,4-DHP derivatives **3**, **6**, and **9,** and 4-(*N*-hexadecylpyridinium)-1,4-DHP derivatives **10** and **11** had zeta-potentials from +26.07 mV (compound **6**) to +62.80 mV (compound **11**), indicating a strongly positive surface charge and confirming the relative electrostatic stability of these nanoparticle solutions. Transmission electron microscopy (TEM) images of nanoaggregates formed by 1,4-DHPs **3** and **11** confirmed liposome-like structures with diameters around 70 to 170 nm. The critical aggregation concentration (CAC) value interval for 4-(*N*-alkylpyridinium)-1,4-DHP was from 7.6 µM (compound **11**) to 43.3 µM (compound **6**). The tested 4-(*N*-alkylpyridinium)-1,4-DHP derivatives were able to quench the fluorescence of the binary 1,6-diphenyl-1,3,5-hexatriene (DPH)—1,2-dipalmitoyl-sn-glycero-3-phosphocholine (DPPC) system, demonstrating hydrophobic interactions of 1,4-DHPs with phospholipids. Thus, 4-(*N*-dodecylpyridinium)-1,4-DHP derivative **3** quenched the fluorescence of the DPH–DPPC system more efficiently than the other 4-(*N*-alkylpyridinium)-1,4-DHP derivatives. Likewise the compound **3**, also 4-(N-dodecylpyridinium)-1,4-DHP derivative **9** interacted with the phospholipids. Moreover, we have established that increasing the length of the alkyl chain at the quaternised nitrogen of the 4-(*N*-alkylpyridinium)-1,4-DHP molecule or the introduction of propargyl moieties in the 1,4-DHP molecule significantly influences the cytotoxicity on HT-1080 (human fibrosarcoma) and MH-22A (mouse hepatocarcinoma) cell lines, as well as the estimated basal cytotoxicity. Additionally, it was demonstrated that the toxicity of the 4-(*N*-alkylpyridinium)-1,4-DHP derivatives on the Gram-positive and Gram-negative bacteria species and eukaryotic microorganism depended on the presence of the alkyl chain length at the *N*-alkyl pyridinium moiety, as well as the number of propargyl groups. These lipid-like compounds may be proposed for the further development of drug formulations to be used in cancer treatment.

## 1. Introduction

Over the past few decades, scientists worldwide have made efforts to expand the discovery and development of a broad range of nanoparticle delivery systems. These studies had been generally supported by the pharmaceutical and food industries as end-users. Liposomes have been widely studied as perspective delivery systems due to their efficiency, biocompatibility, and dual character—their ability to entrap either hydrophobic or hydrophilic drugs, improving their pharmacokinetic and pharmacodynamic properties [1]. Synthetic nanoparticle-forming cationic lipid-like compounds have been developed as delivery agents for the transfer of genetic materials, including plasmid DNA (pDNA) molecules, into cells [2,3,4] and recently also for therapy and diagnostic applications [5,6,7,8]. In general, among the synthetic cationic delivery systems, quaternary ammonium surfactants are more toxic than their analogues, with the cationic charge delocalised in a heterocyclic ring [9].

Diverse groups of nanoparticles have been reported to possess inherent antimicrobial properties [10,11]. Also, the intrinsic antitumor activity of a carrier material based on cationic lipids that have biguanide as a structural fragment have been reported [12]. Enhancement of the therapeutic efficiency of anticancer agents by their incorporation into vitamin E-based delivery systems is well-known approach with great potential [13,14,15]. The elaboration of nanoparticles that have inherent biological properties may have potential in the future development of antimicrobial and/or antitumor therapies [16,17].

**Figure 1:**
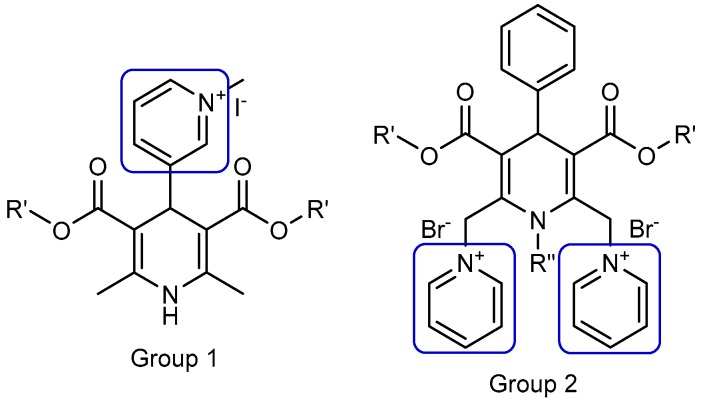
Nanoparticle forming gene delivery systems—amphiphiles on the base of 1,4-dihydropyridine (1,4-DHP) with single (Group 1) and double (Group 2) pyridinium moieties. (Modified from [18]).

Previously, our group elaborated and studied multiple liposomes forming cationic 1,4-dihydropyridine (1,4-DHP) amphiphiles, which were capable of transfecting pDNA into different cell lines in vitro. For an evaluation of the molecule architecture influence on gene delivery properties, various 1,4-DHP amphiphiles containing single or double cationic moieties were designed, synthesised, and studied (Figure 1, groups 1 and 2).

These studies demonstrated that the compounds with double pyridinium moieties (Group 2) showed high transfection efficiencies in vitro and revealed some important structure–activity relationships [18]. After these findings, more detailed studies of 1,4-DHP derivatives containing two pyridinium moieties were performed, and the influence of the cationic part of the molecule [19] and linker [20] on the self-assembling properties and structure–activity relationships were revealed. Additionally, it was established that several representatives of double pyridinium moieties containing compounds (Group 2) possessed antiradical activity [19], and one of them also worked as an antioxidant with the potent reversal of multidrug resistance blocking ability in murine lymphoma cells [21].

As a part of our current research program towards the development of novel non-viral delivery systems possessing biological activities, we continue studies on original single-charged cationic lipids containing a pharmacophore group as putative nanocarriers. Our previous studies have demonstrated that 4-(*N*-dodecylpyridinium)-1,4-DHP derivatives **3** and **6** exhibited calcium antagonistic properties on neuroblastoma SH-SY5Y (IC_50_ about 5–14 µM) and vascular smooth muscle A7R5 (IC_50_ about 0.6–0.7 µM) cell lines [22]. Additionally, the memory-improving anxiolytic effects of 4-(*N*-dodecylpyridinium)-1,4-DHP **3** in transgenic Alzheimer’s disease female mice were described [23], and it was shown that this compound crossed the blood–brain barrier and blocked neuronal and vascular calcium channels [24]. We also found a direct correlation between the length of the alkyl moiety at *N*-quaternised 4-pyridyl-1,4-DHP and improvement of membranotropic effects such as incorporation in the liposomal membranes and bilayer fluidity [25]. Furthermore, studies of the calcium channel antagonist and agonist activities of the 3,5-dipropargylcarbonyl moieties containing 4-(*N*-alkylpyridinium)-1,4-DHPs confirmed that these compounds targeted only calcium channels in vascular smooth muscle cells, and did not affect the calcium channels in cardiac cells [26].

In this work, we have studied in detail the ability to form nanoparticles by cationic 4-(*N*-alkylpyridinium)-1,4-DHP derivatives in aqueous media. Transmission electron microscopy (TEM) images of nanoparticles of selected compounds have been registered. We also established the size distribution and determined the stability of nanoparticles by dynamic light scattering (DLS) measurements. Additionally, critical aggregation concentration (CAC) has been estimated by the DLS technique. Moreover, the cytotoxicity of 4-(*N*-alkylpyridinium)-1,4-DHPs derivatives on HT-1080 (human fibrosarcoma) and MH-22A (mouse hepatocarcinoma) cell lines has been evaluated, and an approximate LD_50_ value has been predicted. Additionally, toxicity on six prokaryotic (bacteria) species and one eukaryotic (yeast) microorganism species have been estimated. The hydrophobic interaction between 4-(*N*-alkylpyridinium)-1,4-DHP derivatives and 1,2-dipalmitoyl-sn-glycero-3-phosphocholine (DPPC) model membranes has been studied. The obtained results will provide a basis for the further understanding of the structure–activity relationships of these compounds.

## 2. Materials and Methods 

### 2.1. 4-(N-Alkylpyridinium)-1,4-Dihydropyridine (1,4-DHP) Derivatives

**Figure 2:**
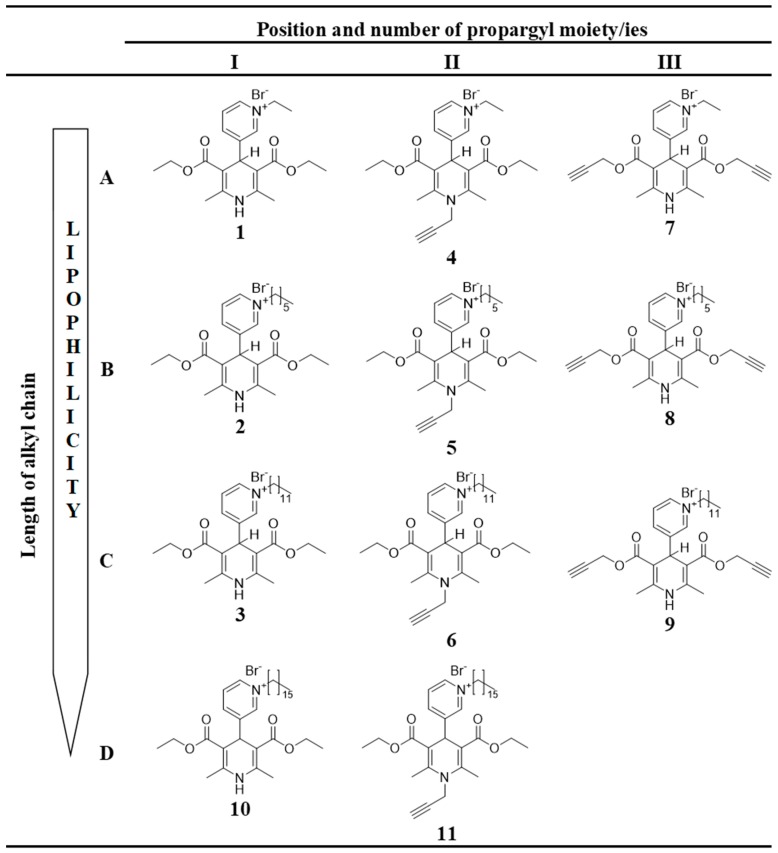
Structures of tested 4-(*N*-alkylpyridinium)-1,4-DHP derivatives **1**–**11** with variation of alkyl chain length (**A**–**D**) and variation of number and position of propargyl moiety (**I**–**III**).

All of the 1,4-DHP derivatives (see Figure 2) provided for the studies have been synthesised in the Laboratory of Membrane active compounds of the Latvian Institute of Organic Synthesis. 4-(*N*-Alkylpyridinium)-1,4-DHP derivatives were obtained according to the already reported methods: 1,4-DHP derivatives **1**–**6**, **10,** and **11** [22] and 1,4-DHP derivatives **7**–**9** [26].

Mass spectra were obtained on a Waters Acquity ultra performance liquid chromatography (UPLC) system (Waters, Milford, MA, USA) connected to a Waters SQ Detector-2 operating in the electrospray ionization (ESI) positive ion mode on a Waters Acquity UPLC^®^ BEH C18 column (1.7 µm, 2.1 × 50 mm) using a gradient elution with MeCN (0.01% CF_3_CO_2_H) in water (0.01% CF_3_CO_2_H) at a flow rate 0.5 mL/min and processed with the Waters MassLynx 4.1 chromatography data system [22,26].

### 2.2. Cell Culture and Measurement of Cell Viability

Tumor cell lines HT-1080 (human connective tissue fibrosarcoma) and MH-22A (mouse hepatocarcinoma) were used. All of the cells were obtained from the American Type Culture Collection ((ATCC,) Rockville, MD, USA).

HT-1080 and MH-22A cells (4 × 10^4^ cell/mL) were seeded in 96-well plates in Dulbecco’s modified Eagle’s medium (DMEM) containing 10% fetal bovine serum and cultivated for 72 h at 37 °C, 5% CO_2_ by exposure to different compounds concentrations (100 μg/mL, 25 μg/mL, 6.25 μg/mL, and 1.56 μg/mL). Cell viability was measured using 3-(4,5-dimethylthiazol-2-yl)-2,5-diphenyl-tetrazolium bromide (MTT) assay. In brief, after incubating with compounds, the culture medium was removed and fresh medium with 0.2 mg/mL MTT was added in each well of the plate. After incubation (3 h, 37 °C, 5% CO_2_), the medium with MTT was removed, and 200 μL of DMSO was added at once to each sample. The samples were tested at 540 nm on a Tecan multiplate reader Infinite1000 (Tecan Austria GmbH, Salzburg, Austria). The IC_50_ was calculated using the program Graph Pad Prism^®^ 3.0.

### 2.3. Basal Cytotoxicity Test 

The neutral red uptake (NRU) assay was performed according to the standard protocol of Stokes [27] modified by NICEATM-ECVAM validation study [28]. The NRU cytotoxicity assay procedure was based on the ability of viable cells to incorporate and bind neutral red, which is a supravital dye.

NIH 3T3 (Mouse Swiss Albino embryo fibroblast) cells (9000 cells/well) were placed into 96-well plates for 24 h in DMEM medium containing 5% fetal bovine serum (FBS). Then, they were exposed to the test compound over a range of seven concentrations (1000 μg/mL, 316 μg/mL, 100 μg/mL, 31 μg/mL, 10 μg/mL, 3.0 μg/mL, and 1.0 μg/mL) for 24 h. Untreated cells were used as a control. After 24 h, the medium was removed from all plates. Then, 250 μL of neutral red (NR) solution was added (0.05 mg/mL NR in DMEM 24 h pre-incubated at 37 °C and then filtered before use through a 0.22-µm syringe filter). Plates were incubated for 3 h, and then, cells were washed three times with phosphate buffered saline (PBS). The dye within viable cells was released by extraction with a mixture of acetic acid, ethanol, and water (1:50:49). The absorbance of neutral red was measured using a spectrophotometer multiplate reader Infinite M1000 (Tecan Austria GmbH, Salzburg, Austria) at 540 nm. The optical density (OD) was calculated using the formula: OD (treated cells) × 100/OD (control cells). The IC_50_ values were calculated using the program Graph Pad Prism^®^ 3.0.

### 2.4. Estimation of LD_50_ from IC_50_ Values

Data from the in vitro tests were used for estimating the starting dose for acute oral systemic toxicity tests in rodent. The in vivo starting dose is an estimated LD_50_ value that is calculated by inserting the in vitro IC_50_ value into a regression formula: log LD_50_ (mM/kg) = 0.439 log IC_50_ (mM) + 0.621 [29]. The value is recalculated to mg/kg, and compounds are evaluated in accordance with four toxicity categories [30]: category 1: LD_50_ ≤ 5 mg/kg (highly toxic); category 2: 5 < LD_50_ ≤ 50 mg/kg (moderately toxic); category 3: 50 < LD_50_ ≤ 300 mg/kg (slightly toxic); category 4: 300 < LD_50_ ≤ 2000 mg/kg (practically non-toxic). Using an alternative in vitro method enables comparing the possible toxicity of new compounds and selecting compounds for further study, which will vastly reduce the number of animal experiments.

### 2.5. Toxicity Screen 

An estimation of the toxicity of 4-(*N*-alkylpyridinium)-1,4-DHP derivatives **3**, **6**, **7**, and **9**–**11** was performed using six prokaryotic (bacteria) and one eukaryotic (yeast) microorganism species according to the procedure elaborated by Suppi et al. [31]. The microorganisms used in the study and their origin were: *Bacillus subtilis* subsp. *spizizenii* (ATCC^®^ 6633™*, Microbiologics^®^, St Cloud, MN, USA), *Escherichia coli* (ATCC^®^ 8739, Microbiologics^®^), *Klebsiella pneumoniae* (Latvian Microorganisms strain collection, LMKK, strain number 535, Riga, Latvia), *Micrococcus luteus* (LMKK, No. 25), *Pseudomonas aeruginosa* (NCTC 12924, Biosciences Ltd., Dublin, Ireland), *Proteus mirabilis* (LMKK, No 590), and *Saccharomyces cerevisiae*, CEN.PK2 strain [32]. 

### 2.6. Microorganism Cultivation and Toxicity Test

For toxicity screening, a compound serial dilution–spot test method as suggested by Suppi et al. was used [31].

Bacteria and yeast cultures were cultivated until their exponential growth phase (optical density max 1); then, 100 µL of cell suspension was taken, washed in deionised (DI) water, and added to serially diluted samples of corresponding 4-(*N*-alkylpyridinium)-1,4-DHPs in DI water. All 1,4-DHPs were tested in five serially diluted concentrations (10 mM, 1 mM, 0.1 mM, 0.01 mM, and 0.001 mM). Test organisms were exposed to 1,4-DHP derivatives in DI water on 96-well microplates (Corning, Corning, NY, USA) at 28 °C for 24 h without shaking in the dark. Then, a 5 µL sample was taken from each well and spotted on yeast extract, bactopeptone, dextrose broth (YPD) (yeast extract 1%, bactopeptone 2%, glucose 2% and agar 2%) plates to test the growth of yeasts and lysogeny broth (LB) (yeast extract 0.5%, bactopeptone 1%, NaCl 0.5% and agar 2%) to test the growth for bacteria. Plates were left in 30 °C for 24 h, and assessed for microbial growth. 

Boundary concentration was defined as the last dilution of 1,4-DHP, which did not fully inhibit the growth of microorganisms (colonies were present). If there were no colonies after incubation with 1,4-DHPs in the last concentration of (0.001 mM), then their boundary concentration was set to 0.0001 mM or 0.1 µM. 

The effects of 4-(*N*-alkylpyridinium)-1,4-DHP derivatives **3**, **6**, **7**, and **9**–**11** on microbial survival (boundary concentrations) were analysed by PCA (Principal Component Analyses) via online ClustVis tool [33].

### 2.7. Phospholipid Binding Assay

Binding studies of the 4-(*N*-alkylpyridinium)-1,4-DHP derivatives **1**–**11** with phospholipid were carried out in Tris-buffered saline (TBS) buffer (10 mM of Tris, 150 mM of NaCl, pH 7.4) by a fluorescence probe technique using a modified protocol described by Ma et al. [34,35]. Briefly, 1,2-dipalmitoyl-sn-glycero-3-phosphocholine (DPPC) vesicles dispersed in TBS buffer were prepared by sonication of the lipid solution using a probe sonifier for 30 min. The concentration of DPPC stock solution was 6 × 10^−5^ M; the concentration of the stock solutions of the 4-(*N*-alkylpyridinium)-1,4-DHP derivatives were 1.5 × 10^−4^ M.

To characterise the hydrophobic interaction between the 1,4-DHP and phospholipids, 1,6-diphenyl-1,3,5-hexatriene (DPH) was used as a fluorescence probe. The lipid-bound DPH shows intense fluorescence at 440 nm (excitation, 368 nm), while the non-bound DPH does not exhibit fluorescence in aqueous solution. DPH was used as a probe to determine the hydrophobic interaction between the esters and the phospholipid. Stock solutions of DPH in acetone (15 × 10^−4^ M) were freshly prepared and kept in the dark and diluted with TBS buffer (pH = 7.4) to concentration.

Mixtures containing 40 µL of DPPC stock solution (6 × 10^−5^ M) and 40 µL of DPH diluted stock solution (15 × 10^−6^ M) were incubated at 50 °C for 0.5 h in the dark; then, an aliquot of 4-(*N*-alkylpyridinium)-1,4-DHP derivatives was added with the following incubation at 50 °C for 0.5 h in the dark, after which samples were cooled to room temperature and the fluorescence intensity was measured by a Tecan multiplate reader Infinite1000 (Tecan Austria GmbH, Salzburg, Austria). The final concentrations of compounds in the sample were: DPPC (2 × 10^−5^ M), DPH (5 × 10^−6^ M), and 1,4-DHP **1**–**5**, **7**, and **9**–**11** (5 × 10^−5^ M).

### 2.8. Self-Assembling Properties by Dynamic Light Scattering Measurements

Samples of compounds for dynamic light scattering (DLS) studies were prepared by thin-film hydration method in an aqueous solution at a concentration of 0.5 mg/mL for compounds **1**–**7**, 0.25 mg/mL for compounds **8**–**10**, and 0.1 mg/mL for compound **11**. A corresponding amount of compounds was weighted in a round-bottom flask and dissolved in chloroform; then, the organic solvent was removed in vacuo, and the residue was dried in high vacuo for 1 h. An appropriate amount of deionised water was added to each flask for the preparation of stock solutions with the above-mentioned concentrations. Samples were prepared by sonication using a bath-type sonicator (Cole Parmer Ultrasonic Cleaner 8891CPX (Vernon Hills, IL, USA)). Samples were sonicated for 60 min at 50 °C.

The DLS measurements of the nanoparticles in an aqueous solution were carried out on a Zetasizer Nano ZSP (Malvern Panalytical Ltd., Malvern, UK) instrument with Malvern Instruments Ltd. Software 7.12, using the following specifications—medium: water; refractive index: 1.33; viscosity: 0.8872 cP; temperature: 25 °C; dielectric constant: 78.5; nanoparticles: liposomes; refractive index of materials: 1.60; detection angle: 173°; wavelength: 633 nm. Data were analysed using the multimodal number distribution software that was included with the instrument. The measurements were performed in triplicate in order to check their reproducibility.

### 2.9. Determination of CAC

The critical aggregation concentrations (CAC) of 4-(*N*-alkylpyridinium)-1,4-DHP derivatives were determined in aqueous media using a Zetasizer Nano S90 (Malvern Panalytical Ltd., Malvern, UK) instrument with Malvern Instruments Ltd. Software according to procedure described by Topel et al. and modified by our group [19,36]. Briefly, as stock solutions of tested compounds were used, samples were prepared for self-assembling experiments. All of the subsequent samples were prepared starting from the concentrated stock solution, which was subjected to a serial two-fold dilution each time with water. The intensity values of scattered light (kcps) as a function of concentration of amphiphiles were analysed. The scattering intensities detected for amphiphile concentrations below CAC have an approximately constant value corresponding to water. The intensity starts to show a linear increase with concentration at the CAC, since the amount of nanoparticles increases in the solution. The intersection of the best-fit lines drawn through the data points is the preliminary CAC value of the compounds.

### 2.10. Transmission Electron Microscopy (TEM) 

The morphology of nanoparticles formed by selected 4-(*N*-alkylpyridinium)-1,4-DHP derivatives **3** and **11** (0.15 mM) was studied by TEM. Negative stained TEM samples were prepared using the side blotting method. The sample solution was placed on a carbon-coated grid (Agar AGS160-4); excess solution was drained with filter paper, and then it was negatively stained with 2% uranyl acetate, which was freshly prepared and filtered with a 0.22-µm filer. TEM measurements were performed using Tecnai G2 F20 (FEI, Hillsboro, OR, USA) microscope at 60 kV.

### 2.11. Statistical Analysis

Results are expressed as mean standard deviation (SD). All of the experiments were performed in triplicate. 

## 3. Results and Discussion

### 3.1. 4-(N-Alkylpyridinium)-1,4-Dihydropyridine Derivatives

The synthesis of selected 4-(*N*-alkylpyridinium)-1,4-dihydropyridine derivatives **1**–**11** differing in alkyl chain length and propargyl moiety/ties number and position was carried out by previously described methods [22,26]. Briefly, the 1,4-DHP derivatives were synthesised via typical synthetic routes, including the classical Hantzsch method [37], which involved the one-pot cyclocondensation of the corresponding esters of acetoacetic acid, and the corresponding aldehyde and ammonia or ammonium acetate as a nitrogen source in ethanol under reflux. The *N*-propargyl-substituted 1,4-DHP derivatives were obtained by analogy with the synthesis of other *N*-substituted 1,4-DHP ones via a modified Hantzsch-type cyclisation using propargyl amine hydrochloride as a nitrogen source instead of ammonia and pyridine as a solvent under reflux. The synthesis of all the 4-pyridinium moieties containing 1,4-DHP derivatives were performed by the alkylation of 4-pyridyl-1,4-DHP derivatives with the corresponding alkyl bromides in acetone under reflux. The typical procedure for the quaternisation of pyridyl-1,4-DHP derivatives was performed in analogy with the procedure described by Makarova et al. [38,39].

In order to evaluate the influence of the pharmacophore group on the structure–activity relationship, 11 1,4-DHP derivatives were divided into two groups considering structure elements (see Figure 2):

1. 1,4-DHP derivatives with different lengths of alkyl moieties at the quaternised nitrogen atom at the position 4 of the 1,4-DHP ring (Figure 2, rows A–D):
  1.1. 4-(*N*-Ethylpyridinium)-1,4-DHP derivatives (compounds **1**, **4**, **7**);
  1.2. 4-(*N*-Hexylpyridinium)-1,4-DHP derivatives (compounds **2**, **5**, **8**);
  1.3. 4-(*N*-Dodecylpyridinium)-1,4-DHP derivatives (compounds **3**, **6**, **9**);
  1.4. 4-(*N*-Hexadecylpyridinium)-1,4-DHP derivatives (compounds **10**, **11**).
2. 1,4-DHP derivatives without or with shifted propargyl moiety/ies (Figure 2, columns I, II, III):
  2.1. 1,4-DHP derivatives without propargyl moiety/ies (compounds **1**–**3** and **10**);
  2.2. Propargyl moiety at the position 1 of the 1,4-DHP ring (1-propargyl-4-(*N*-alkylpyridyl)-1,4-DHP; compounds **4**–**6**, **11**);
  2.3. Propargyl moieties at the positions 3 and 5 of the 1,4-DHP ring (bispropargyl 4-(*N*-alkylpyridinium)-1,4-DHP 3,5-dicarboxylates; compounds **7**–**9**).

The purities of the studied compounds were at least 97% according to high-performance liquid chromatography (HPLC) data. The detailed synthetic procedures, yields of products, nuclear magnetic resonance (NMR) spectrum data for 4-(*N*-alkylpyridinium)-1,4-dihydropyridine derivatives **1**–**11** were described by Rucins et al. [22,26].

### 3.2. Estimation of LD_50_ from IC_50_ Values

Antiproliferative activity and also a potential toxic effect were evaluated for 4-(*N*-alkylpyridinium)-1,4-DHP derivatives **1**–**9**. Currently, it is recommendable to start toxicology studies with preliminary in vitro estimation before using animal models [40]. It has been proposed that the equation from the correlation of IC_50_ (the concentration of a substance that causes 50% toxicity in vitro) could be applied to estimate unknown LD_50_ values for a new compound from IC_50_ values measured as basal cytotoxicity in vitro. This estimated LD_50_ gives prior information regarding compound properties and would be used to select promising compounds and a starting dose for in vivo experiments. 

The evaluation of cytotoxicity of 4-(*N*-alkylpyridinium)-1,4-DHP derivatives **1**–**9** in vitro was assessed using the colorimetric 3-(4,5-dimethylthiazol-2-yl)-2,5-diphenyltetrazolium bromide (MTT) assay on two monolayer tumor cell lines, namely HT-1080 (human fibrosarcoma) and MH-22A (mouse hepatocarcinoma). Additionally, the compound influence on “normal” mouse fibroblasts (NIH 3T3) was estimated for the studies of structure–activity relationships and exploration of the effect of substituents. The results are presented in Table 1. 

The obtained data showed that 4-(*N*-ethylpyridinium)-1,4-DHP derivatives **1**, **4**, and **7** did not demonstrate any cytotoxic effect on tumor HT-1080 and MH-22A cell lines, and their estimated toxicity LD_50_ was defined as practically non-toxic (basal cytotoxicity LD_50_ ≥2000 mg/kg); LD_50_ values for compounds **1**, **4**, and **7** are 2548, 2280 and >2710, respectively. In contrast, 4-(*N*-hexylpyridinium)-1,4-DHP derivatives **2**, **5**, and **8** and 4-(*N*-dodecylpyridinium)-1,4-DHP derivatives **3**, **6**, and **9** possessed cytotoxicity on tumor cell lines (IC_50_ 1–80 μM) and their estimated LD_50_ was defined as slightly toxic (183 mg/kg and 300 mg/kg for compound **6** and **9**, respectively) or non-toxic (1404, 692, 669, and 1165 for 1,4-DHP derivatives **2**, **3**, **5**, and **8**, respectively). Very pronounced cytotoxicity was observed for 1-propargyl substituted 4-(*N*-hexylpyridinium)-1,4-DHP derivative **5** (IC_50_ 5 μM in the HT-1080) and also for 1-propargyl substituted 4-(*N*-dodecylpyridinium)-1,4-DHP derivative **6** and bispropargyl 4-(*N*-dodecylpyridinium)-1,4-DHP 3,5-dicarboxylate **9**, where IC_50_ values were in the range of 1 to 4 μM in the both cell lines. Meanwhile, IC_50_ values in the both cell lines were in the range of 15 to 80 μM for 4-(*N*-hexylpyridinium)-1,4-DHP **2** and 4-(*N*-dodecylpyridinium)-1,4-DHP **3**, both compounds without propargyl moieties, bispropargyl 4-(*N*-hexylpyridinium)-1,4-DHP 3,5-dicarboxylate **8**, and only in the MH-22A cell line for 1-propargyl substituted 4-(*N*-hexylpyridinium)-1,4-DHP derivative **5**. Our observation suggests that increasing the length of the alkyl chain from ethyl to dodecyl at the quaternised nitrogen atom at position 4 of the 1,4-DHP significantly increases the cytotoxicity as well as the estimated basal cytotoxicity of compounds (**1** versus **2** versus **3** and **4** versus **5** versus **6** and **7** versus **8** versus **9**: 2548/1404/692 and 2280/669/183 mg/kg and >2700/1165/300 mg/kg, respectively (Table1)). Additionally, it was observed that the introduction of propargyl moiety/moieties in the 1,4-DHP molecule also increased the basal cytotoxicity of 4-(*N*-hexylpyridinium)-1,4-DHP and 4-(*N*-dodecylpyridinium)-1,4-DHP derivatives, especially at position 1 of the 1,4-DHP cycle (**2** versus **5** and **8**; **3** versus **6** and **9**: 1404/669 and 1165 mg/kg; 692/183 and 300 mg/kg, respectively (Table 1)). Generally, it can be concluded that increasing the length of the alkyl chain from ethyl to dodecyl at the quaternised nitrogen atom at position 4 of the 1,4-DHP ring or the introduction of propargyl moiety/moieties into the 1,4-DHP molecule influences both the cytotoxicity against cancer cells and also the estimated basal cytotoxicity of tested 4-(*N*-alkylpyridinium)-1,4-DHP derivatives.

### 3.3. Toxicity Test

Several different species of microorganisms were chosen to evaluate toxicity of 4-(*N*-alkylpyridinium)-1,4-DHP derivatives **3**, **6**, **7**, and **9**–**11** on microorganisms according to the procedure elaborated by Suppi et al. [31]. The panel of microorganisms was composed based on an ecotoxicological review by Egorova and Ananikov [38]. It included representatives of Gram-positive bacteria species (*B. subtilis* and *M. luteus*) and representatives of Gram-negative bacteria species (*E. coli*, *P. miriabilis*, *P. aeroginosa,* and *K. pneumoniae*) and one example of eukaryotic microorganism (*S. cerevisiae*). The obtained results (see Table S1 in the Supplementary Material) regarding the effect of 4-(*N*-alkylpyridinium)-1,4-DHP derivatives **3**, **6**, **7**, and **9**–**11** on microbial survival were analysed by Principle Component Analyses (PCA) using the online ClustVis tool [33]. PCA is a mathematical procedure that reduces the dimensionality of large datasets, increasing their interpretability while at the same time minimizing information loss. Currently, PCA is one of the most important and powerful methods for standard statistical tests in biology, medicine, and other fields using statistical tests [41,42]. In our studies, PCA were used to evaluate how the influence of the molecular parameters (the presence of a propargyl group and/or length of the alkyl chain) of the 4-(*N*-alkylpyridinium)-1,4-DHP derivatives affected their toxicity in the tested microorganism panel. The boundary concentrations from the chosen 1,4-DHP derivatives that were tested in all the microorganisms were collected in a score matrix (Table S1 in the Supplementary Material), and the obtained data resulted in a four-dimension matrix (number of propargyl groups; side chain length; microorganism; boundary concentration).

**Figure 3:**
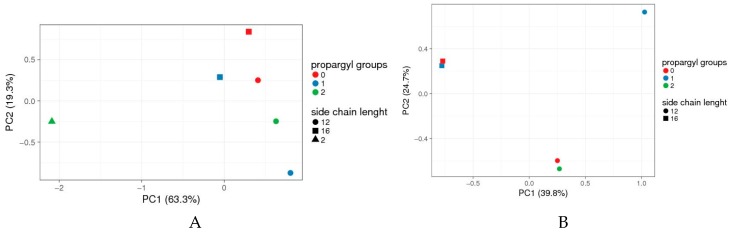
Principal Component Analyses (PCA) data of 4-(*N*-alkylpyridinium)-1,4-DHP toxicity when analysed by compound boundary concentrations. (**A**) Full set of compounds, (**B**) 1,4-DHP **7** omitted. Boundary concentrations from all tested 1,4-DHP derivatives tested in all microorganisms were collected in a score matrix (Table S1 in Supplementary Material); the results formed a four-dimension matrix (number of propargyl groups; side chain length; microorganism; boundary concentration). To find out which of the parameters (number of propargyl groups and/or side chain length) has the most significant impact on the compound’s toxicity, the “artificial” set of variables that would help to visualise the main effects of data variance across a two-dimensional (2D) plot was sought. The data were analysed and visualised by online tool ClustVis [33]. Vector scaling is applied to rows; the Nonlinear Iterative Partial Least Squares (NIPALS) algorithm is used to calculate principal components [42]. The scores of individual compound’s toxicities are plotted using the first two principal components, which explain most of the variance (PC1 63.3% and PC2 19.3% for A, and 39.8% and 24.7% for B). Green triangle: 4-(*N*-ethylpyridinium)-1,4-DHP **7**.

The 4-(*N*-ethylpyridinium)-1,4-DHP **7** did not affect the survival of microorganisms in the whole range of the concentrations tested (10 to 0.001 mM). Similarly, this derivative turned out to be non-toxic to the tested cancer cell lines also. On the contrary, other tested 1,4-dihydropyridine derivatives containing *N*-dodecylpyridinium (compounds **3**, **6,** and **9**) or *N*-hexadecylpyridinium (compounds **10** and **11**) moieties were toxic at various degrees, affecting the viability of the microorganisms. A similar relationship was observed also for ionic liquids (ILs): the increase in the length of the alkyl chain on the ILs’ heterocyclic ring significantly increased their toxicity in most test systems [43,44]. To evaluate the patterns of 4-(*N*-alkylpyridinium)-1,4-DHP alkyl chain effect on the survival of microorganisms PCA were carried out using each compound boundary concentrations for each microorganism. The obtained data are presented in Figure 3.

Data for the set of tested 4-(*N*-alkylpyridinium)-1,4-DHP derivatives and their toxicities (Table S1 in Supplementary Material), indicated that compounds were rather scattered across a two-dimensional (2D) plot with no pattern clustering neither with respect to the number of propargyl groups and alkyl chain length at alkylpyridinium moiety/ies. The toxicity of 4-(*N*-ethylpyridinium)-1,4-DHP **7** substantially differs from the toxicities of *N*-dodecylpyridinium (compounds **3**, **6**, and **9**) or *N*-hexadecylpyridinium (compounds **10** and **11**) derivatives (Figure 3A). However, if compound **7** was omitted from PCA analyses, it can be concluded that 4-(*N*-alkylpyridinium)-1,4-DHPs were grouped mainly based on their *N*-alkyl chain length rather than the number of propargyl groups (Figure 3B).

Therefore we assume that the toxicity of the compound depends on the *N*-alkyl chain length at the pyridinium moiety and/or the number of propargyl groups.

According to the literature data in microorganism assays, the tested toxicity of various cationic surfactants that are capable of forming nanoaggregates were dependent on the alkyl chain length. Thus, the values of IC_50_ for liposomes formed by hexadecyltrimethylammonium bromide and didodecyldimethylammonium bromide were higher than the IC_50_ values for liposomes formed by dioctadecyldimethylammonium bromide [45]. In our experiments, it was demonstrated that 4-(*N*-dodecylpyridinium)-1,4-DHPs (compounds **3**, **6**, and **9**) were more toxic than other 4-(*N*-alkylpyridinium)-1,4-DHPs, such as 4-(*N*-ethylpyridinium)-1,4-DHP **7** and 4-(*N*-hexadecylpyridinium)-1,4-DHPs (compounds **10** and **11**).

### 3.4. Phospholipid Binding Assay

Lipid bilayers are essential in the regulation of in vivo barriers, and surfactants are known to influence their organization and permeability [46]. 1,2-Dipalmitoyl-sn-glycero-3-phosphocholine (DPPC) is a major membrane-forming phospholipid [47] in cell membranes, and is also widely used as a model membrane to provide valuable information regarding the interaction of cell membranes and lipids [48] and other various surfactants as antibacterial agents [49]. Therefore, in order to estimate the potential impact of 4-(*N*-alkylpyridinium)-1,4-DHP derivatives on microorganisms, the binding assay of DPPC with the cationic 1,4-DHP derivatives were performed.

DPPC bound to 1,6-diphenyl-1,3,5-hexatriene (DPH) shows enhanced fluorescence intensity at 440 nm (excited at 368 nm). According to the literature data, DPH interacts with DPPC through hydrophobic interaction, and compounds are also capable of binding with the phospholipids through hydrophobic interactions to quench the fluorescence of the lipid–DPH solution due to the competitive binding mechanism [34]. In this work, DPH was used as a fluorescent probe to characterise the interaction between the phospholipids and 4-(*N*-alkylpyridinium)-1,4-DHP derivatives. 

**Figure 4:**
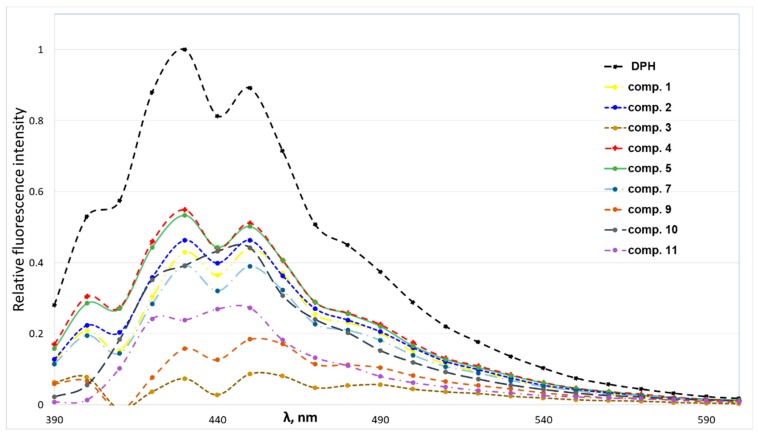
Fluorescence intensity spectra of solutions containing 1,2-dipalmitoyl-sn-glycero-3-phosphocholine (DPPC) (2 × 10^−5^ M) and 1,6-diphenyl-1,3,5-hexatriene (DPH) (5 × 10^−6^ M) in the absence or presence of 4-(*N*-alkylpyridinium)-1,4-dihydropyridines **1**–**5**, **7** and **9**–**11** (5 × 10^−5^ M).

According to obtained data (Figure 4), 4-(*N*-alkylpyridinium)-1,4-DHP derivatives quenched the fluorescence of the DPPH–DPH solution. The decrease of sample fluorescence in the presence of 1,4-DHPs indicated that 4-(*N*-alkylpyridinium)-1,4-DHPs were bound to the model membrane near the DPH binding sites. The 4-(*N*-alkylpyridinium)-1,4-DHP derivatives were able to quench the fluorescence of the binary DPH–DPPC, confirming that the tested 4-(*N*-alkylpyridinium)-1,4-DHP derivatives **1**–**5** and **8**–**11** interact with the phospholipids via hydrophobic interactions in the order of **3** > **9** > **11** > **7** ≥ **1** ≥ **10** ≥ **2** > **5** ≥ **4**. We failed to obtain comparable data for the influence of 4-(*N*-dodecylpyridinium)-1,4-DHP derivative **6** interaction on the binary DPH–DPPC system; therefore, the data for compound **6** was not included in Figure 4. In the case of 4-(*N*-dodecylpyridinium)-1,4-DHP derivative **3**, the lowest relative fluorescence intensity was demonstrated, which confirmed the ability of this compound to quench the fluorescence of the DPH–DPPC system more efficiently than the other 4-(*N*-alkylpyridinium)-1,4-DHPs. Also, the ability of the 4-(*N*-dodecylpyridinium)-1,4-DHP derivative **9** to quench the fluorescence of the DPH–DPPC system can be compared with the one obtained for compound **3**. Our previous data showed that 4-(*N*-hexadecylpyridinium)-1,4-DHP derivative **10** and structurally related compounds caused the remarkable release of calcein from DPPC liposomes and induced the hemolysis of human erythrocytes, which confirmed correlation between the length of the alkyl moiety at *N*-quaternised 4-pyridyl-1,4-DHP and the improvement of membranotropic effects such as incorporation in the liposomal membranes and bilayer fluidity [25].

### 3.5. Self-Assembling Properties

The aim of this work was to characterise the self-assembling properties of 4-(*N*-alkylpyridinium)-1,4-DHP derivatives **1**–**11** as synthetic lipid-like compounds and study the formation of nanoparticles for the evaluation of their potential applications as nanocarriers. The detailed characterisation of nanosystems formed by lipid-like 1,4-DHPs is essential for the understanding of structure–activity relationships, which can be used for further rational design principles of delivery systems. According to our experience with dynamic light scattering (DLS) measurements, samples of dicationic 1,4-DHP amphiphiles (Figure 1, Group 2) and their structural analogues have to be prepared by dispersing a fixed amount of compound in an aqueous solution by sonication using a bath-type [20] or a probe-type sonicator [19].

However, in the case of 4-(*N*-alkylpyridinium)-1,4-DHP derivatives **1**–**11**, both previously mentioned sample preparation methods did not work properly, and homogenous solutions of the tested compounds at required concentrations were not obtained. Therefore, for DLS measurements, the aqueous solutions of 4-(*N*-alkylpyridinium)-1,4-DHP derivatives **1**–**11** were prepared by the thin-film hydration method. The final concentrations of the obtained samples of 1,4-DHP derivatives **1**–**7** were 0.5 mg/mL, and due to poor solubility of the other compounds, it was 0.25 mg/mL for compounds **8**–**10**, and 0.1 mg/mL for compound **11**. The hydrodynamic diameters, polydispersity index (PDI), zeta-potential, and stability of nanoparticles formed by 4-(*N*-alkylpyridinium)-1,4-DHP derivatives **1**–**11** in aqueous medium were determined by the DLS method, and the data are presented in Table 2. The first set of DLS measurements represents the results obtained for freshly prepared samples. It was demonstrated that 4-(*N*-alkylpyridinium)-1,4-DHP derivatives **1**–**4**, **6**–**8**, and **10** formed nanoparticles mainly as one population (over 95%), while 4-(*N*-alkylpyridinium)-1,4-DHP derivatives **9** and **11** formed two main populations at various ratios (Table 2), and 4-(*N*-hexylpyridinium)-1,4-DHP derivative **5** formed one main population (78%) and various smaller populations (up to 1–2%) (Table 2).

Additionally, the results showed a rather broad particle size distribution for compounds **2**, **5**, **7**, and **8**, possessing PDIs for the first set samples of 0.50, 0.72, 0.90, and 0.60, respectively. The obtained data for all the 4-(*N*-hexylpyridinium)-1,4-DHP derivatives **2**, **5**, and **8** and bispropargyl 4-(*N*-ethylpyridinium)-1,4-DHP 3,5-dicarboxylate **7** confirmed the evidence of highly heterogeneous samples from both of the measurement sets. The evaluation of nanoparticles formed by 4-(*N*-alkylpyridinium)-1,4-DHP derivatives **2**, **5**, **7**, and **8** with broad particle size distribution will not be discussed further here, because these compounds are not perspective. 

The PDI value was 0.36 for a freshly prepared sample of bispropargyl 4-(*N*-dodecylpyridinium)-1,4-DHP 3,5-dicarboxylate **9**; however, the sample contained two main populations of nanoparticles with mean diameters 65 nm and 297 nm in the ratio 1:3 (Table 2). The similar PDI value of 0.37 was observed also for a freshly prepared sample of *N*-propargyl moiety containing 4-(*N*-hexadecylpyridinium)-1,4-DHP derivative **11**. This sample contained two main populations of nanoparticles with mean diameters of 145 nm and 51 nm in the ratio of 10:1 (Table 2). The most homogeneous particles were formed by 4-(*N*-alkylpyridinium)-1,4-DHP derivatives **1**, **3**, **4**, **6**, and **10** with PDI values of 0.18, 0.14, 0.16, 0.15, and 0.10, respectively. 

Values of the zeta-potential of nanoparticles formed by 4-(*N*-alkylpyridinium)-1,4-DHP derivatives **1**–**11** were determined by DLS (Table 2). The obtained data indicated that nanoparticles formed by 4-(*N*-dodecylpyridinium)-1,4-DHPs (compounds **3**, **6**, and **9)** and 4-(*N*-hexadecylpyridinium)-1,4-DHPs (compounds **10** and **11)** had zeta-potentials from +26.07 mV (compound **6**) to +62.80 mV (compound **11**), indicating a strongly positive surface charge (Table 2), while the values of the zeta-potential of nanoparticles formed by 4-(*N*-ethylpyridinium)-1,4-DHPs (compounds **1**, **4**, and **6**) and 4-(*N*-hexylpyridinium)-1,4-DHPs (compounds **2** and **5**) were slightly negative (interval from −0.78 mV (compound **7**) until −9.96 mV (compound **5**). Zeta-potentials over >±20 mV confirmed that the formed nanoparticle solutions were also relatively electrostatically stable [50].

For the evaluation of the stability of nanoparticles, the second set of measurements was undertaken after two weeks of storage (samples were stored at r.t. between sets). It was found that the samples of 4-(*N*-ethylpyridinium)-1,4-DHP derivatives **1** and **4** and 4-(*N*-dodecylpyridinium)-1,4-DHP derivative **9** lost their homogeneity after two weeks of storage (Table 2). For these compounds, the PDI values were increased to 0.40, 0.41, and 0.57, respectively, compared to the initial values of 0.18, 0.16, and 0.36. The main point to be noted is that the measurements demonstrated the homogeneity of the particles, which were composed of 4-(*N*-dodecylpyridinium)-1,4-DHP derivatives **3**, **6** and 4-(*N*-hexadecylpyridinium)-1,4-DHP derivative **10**. Thus, after two weeks of storage, the PDI values were 0.17, 0.20, and 0.25, respectively. The sample for 4-(*N*-hexadecylpyridinium)-1,4-DHP derivative **11** maintained constant characteristic parameters: PDI values 0.37 and 0.33, and mean diameters of 145 nm (91%) and 143 (96%), respectively. The obtained data confirmed the stability of nanoparticles formed by 1,4-DHP derivatives **3**, **6**, **10**, and **11** after two weeks of storage.

For self-assembling compounds, the concentration above which micelles and other nanoparticles are formed, which is called the critical aggregation concentration, is an important property. In this study, CAC was determined for 4-(*N*-alkylpyridinium)-1,4-DHP derivatives **1**–**7** and **9**–**11** by the DLS measurement. A representative example of the determination of CAC for 1,4-DHP **11** is presented at Figure S1 in the Supplementary Material. The determined CAC values for tested 4-(*N*-alkylpyridinium)-1,4-DHP derivatives **3**, **6**, and **9**–**11** were in intervals from 7.6 µM (compound **11**) to 43.3 µM (compound **6**) (Table 2). CAC values for 4-(*N*-ethylpyridinium)-1,4-DHP derivatives **1**, **4**, **7**, and 4-(*N*-hexylpyridinium)-1,4-DHP derivatives **2** and **5** cannot be determined clearly. An unclear CAC and negative and low zeta-potential value (interval from −0.78 mV until −9.96 mV) of particles formed by 4-(*N*-alkylpyridinium)-1,4-DHP derivatives **1**, **2**, **4**, **5**, and **7** also confirmed the weak stability of the nanoparticles formed by these compounds. Our previous data showed that the CAC values of double pyridinium moieties containing 1,4-DHP derivatives (Figure 2, Group 2) were in the range of 7 to 35 µM, depending on nature of substituents [19,51]. The obtained data of CAC has indicated that the increase of the *N*-alkyl chain length at the pyridinium moiety in the 1,4-DHP molecule decreased the CAC value. For 4-(*N*-dodecylpyridinium)-1,4-DHP derivative **3**, the CAC value is comparable with the concentration of a substance that causes 50% toxicity in vitro (IC_50_) on HT-1080 and MH-22A cell lines. It suggests that in this concentration, the cytotoxicity was estimated as the cytotoxicity of nanoparticles.

The obtained results confirmed that the presence of a cationic charge and *N*-dodecylpyridinium or *N*-hexadecylpyridinium moiety at the 1,4-DHP cycle was essential for the formation of stable nanoparticles (**3** versus **1** and **2**; **6** versus **4** and **5**) with mean diameter values in the rage of 124 to 184 nm (4-(*N*-dodecylpyridinium)-1,4-DHPs **3** and **6**); 145 to 221 nm (4-(*N*-hexadecylpyridinium)-1,4-DHPs **10** and **11**) (Table 2). Additionally, the introduction of propargyl moieties at positions 3 and 5 of the 1,4-DHP ring decreased the homogeneity and stability of the formed nanoparticles by bispropargyl 4-(*N*-dodecylpyridinium)-1,4-DHP 3,5-dicarboxylate **9** if compared to 4-(*N*-dodecylpyridinium)-1,4-DHP **3** and 1-propargyl-(*N*-dodecylpyridinium)-1,4-DHP **6** (Table 2).

### 3.6. Transmission Electron Microscopy (TEM)

**Figure 5:**
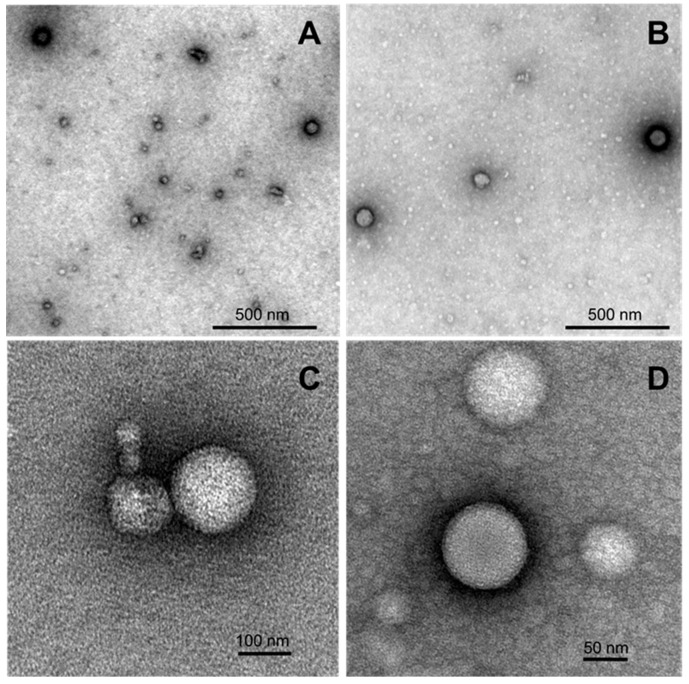
TEM images of formed nanostructures for the samples of 4-(*N*-dodecylpyridinium)-1,4-DHP **3** (**A**,**C**) and 1-propargyl-4-(*N*-hexadecylpyridinium)-1,4-DHP **11** (**B**,**D**) adsorbed to carbon-coated grids and negatively stained with freshly prepared 2% uranyl acetate aqueous solution. The stock samples were prepared by the thin-film hydration method following sonication in an aqueous solution at 1,4-DHP concentrations of 0.5 mM (compound **3**) and 0.15 mM (compound **11**).

Morphology of the samples of nanoaggregates formed by selected 4-(*N*-dodecylpyridinium)-1,4-DHP **3** and 1-propargyl-4-(*N*-hexadecylpyridinium)-1,4-DHP **11** was studied using transmission electron microscopy (TEM) techniques. Representative TEM images of samples are given in Figure 5. 

According to the obtained data, both samples contained spherical particles that were almost spherical in shape, with the diameters ranging in the interval from 70 to 170 nm. The obtained TEM data is also in agreement with the DLS measurement results, which enable making an assumption that the nanoparticles that are formed by selected 4-(*N*-dodecylpyridinium)-1,4-DHP **3** and 4-(*N*-hexadecylpyridinium)-1,4-DHP **11** are liposome-like structures.

## 4. Conclusions

The obtained results reveal that a variation of alkyl moiety lengths for the quaternised nitrogen atom at position 4 of the 1,4-DHP cycle and the propargyl moiety number and position in the 4-(*N*-alkylpyridinium)-1,4-DHP molecule strongly affects the self-assembling properties of compounds and characteristic parameters, and the stability of formed nanoparticles, as well as the toxicity of tested lipid-like 1,4-DHP derivatives. It is shown that 4-(*N*-ethylpyridinium)-1,4-DHP derivatives **1**, **4**, and **7** do not exhibit a cytotoxic effect on tumor HT-1080 and MH-22A cell lines. The estimated basal cytotoxicity (LD_50_) of 4-(*N*-ethylpyridinium)-1,4-DHP derivatives is defined as practically non-toxic. Meanwhile, 4-(*N*-hexylpyridinium)-1,4-DHPs (**2**, **8**, and **8**) and 4-(*N*-dodecylpyridinium)-1,4-DHP derivatives (**3**, **6**, and **9**) possess cytotoxicity on tumor cell lines (IC_50_ 1–80 μM), and the estimated basal cytotoxicity (LD_50_) of the compounds is defined as slightly toxic or non-toxic. Also, the toxicity evaluated on Gram-positive and Gram-negative bacteria species and eukaryotic microorganism demonstrated the difference between 4-(*N*-ethylpyridinium)-1,4-DHP **7** and longer alkyls, such as 4-(*N*-dodecylpyridinium)-1,4-DHP derivatives **3**, **6**, and **9** and 4-(*N*-hexadecylpyridinium)-1,4-DHP derivatives **10** and **11**. 

It can be concluded that the presence of *N*-dodecylpyridinium moiety and *N*-hexadecylpyridinium moiety at the 1,4-DHP cycle is essential for the formation of stable nanoparticles with mean diameter values in the range of 124 to 221 nm. However, the introduction of propargyl moieties at positions 3 and 5 of the 1,4-DHP ring decreases the stability and homogeneity of the formed nanoparticles. The obtained TEM data enables the suggestion that the nanoparticles formed by the selected 4-(*N*-dodecylpyridinium)-1,4-DHP **3** and 4-(*N*-hexadecylpyridinium)-1,4-DHP **11** are liposome-like structures.

The 4-(*N*-alkylpyridinium)-1,4-DHP derivatives quenched the fluorescence of the DPH of the binary DPH–DPPC system, confirming hydrophobic interaction with the phospholipids. 4-(*N*-Dodecylpyridinium)-1,4-DHP derivatives **3** and **9** quenched the fluorescence of the DPH–DPPC system more efficiently than the other 4-(*N*-alkylpyridinium)-1,4-DHPs. These findings are highly important, since 4-(*N*-dodecylpyridinium)-1,4-DHP **3** has already been reported to efficiently cross the blood–brain barrier, block brain calcium channels, and improve memory by enhancing the GABAergic and synaptic plasticity processes as well as influencing the levels of brain proteins, which contributes to synaptic plasticity [24]. After more detailed studies, some of these lipid-like 4-(*N*-dodecylpyridinium)-1,4-DHP derivatives can be proposed for use in cancer therapy as nanocarriers with intrinsic cytotoxic activity.

## Figures and Tables

**Table 1:** Comparison of cytotoxicity, estimated basal cytotoxicity of 4-(*N*-alkylpyridinium)-1,4-DHP derivatives **1**–**9**.

Comp.	Cytotoxicity IC_50_, μM		Basal Cytotoxicity Estimated ^a^ LD_50_	
	HT-1080	MH-22A	mM/kg	mg/kg
**1**	ne	ne	5.8 ± 0.3	2548
**2**	26 ± 2	67 ± 4	2.8 ± 0.3	1404
**3**	37 ± 2	15 ± 1	1.2 ± 0.4	692
**4**	ne	ne	4.8 ± 0.1	2280
**5**	5 ± 0.3	16 ± 1	1.3 ± 0.4	669
**6**	4 ± 0.4	1 ± 0.2	0.3 ± 0.1	183
**7**	ne	ne	>6	>2710
**8**	76 ± 8	80 ± 5	2.2 ± 0.4	1165
**9**	2 ± 0.2	1 ± 0.2	0.5 ± 0.05	300

^a^ Estimated LD_50_ was calculated based on IC_50_ (mM) value from NIH 3T3 cells Neutral Red Uptake assay (NRU) assay using the regression formula: log LD_50_ (mM/kg) = 0.439 log IC_50_ (mM) + 0.621 [29]. Ne—no effect in the tested concentration.

**Table 2:** Values of critical aggregation concentration (CAC), the mean diameter (D_mean_), zeta-potential, and polydispersity index (PDI) of nanoparticles formed by 4-(*N*-alkylpyridinium)-1,4-DHP derivatives **1**–**11** obtained by dynamic light scattering (DLS) measurements. Final compound concentrations were 0.5 mg/mL for compounds **1**–**7**, 0.25 mg/mL for compounds **8**–**10**, and 0.1 mg/mL for compound **11**. The mean diameter (D_mean_) depicts the hydrodynamic diameter of the main population of nanoparticles in the tested sample; the PDI value describes polydispersity of the sample; the zeta-potential gives information about the surface charge of nanoparticles. CAC is the concentration above which micelles and other nanoparticles are formed.

Comp.	Mw ^a^	CAC, µM	Zeta-Pot ^b^. ± SD, mV	PDI ± SD		D_mean_ ± SD, nm (%)	
				1 Set ^c^	2 Set ^d^	1 Set ^c^	2 Set ^d^
**1**	359	-	−8.43 ± 2.14	0.18 ± 0.01	0.40 ± 0.16	58 ± 24 (98)	195 ± 75 (59) 45 ± 13 (41)
**2**	415	-	−11.39 ± 1.85	0.50 ± 0.10	0.91 ± 0.10	142 ± 24 (95)	225 ± 25 (100)
**3**	499	9.3	+57.73 ± 2.30	0.14 ± 0.01	0.17 ± 0.02	124 ± 51 (100)	140 ± 64 (100)
**4**	397	-	−5.47 ± 1.97	0.16 ± 0.02	0.41 ± 0.07	79 ± 29 (99)	238 ± 76 (78) 55 ± 19 (22)
**5**	453	-	−9.96 ± 0.72	0.72 ± 0.32	0.59 ± 0.01	513 ± 55 (78)	503 ± 66 (100)
**6**	537	43.3	+26.07 ± 0.78	0.15 ± 0.02	0.20 ± 0.01	154 ± 64 (100)	184 ± 97 (100)
**7**	379	-	−0.78 ± 0.26	0.90 ± 0.03	0.73 ± 0.09	258 ± 33 (100)	185 ± 24 (100)
**8**	435	-	-	0.60 ± 0.06	0.45 ± 0.05	325 ± 50 (100)	330 ± 49 (100)
**9**	519	9.3	+32.00 ± 4.81	0.36 ± 0.08	0.57 ± 0.05	65 ± 21 (73) 297 ± 78 (26)	415 ± 88 (98) 58 ± 7 (2)
**10**	555	28.2	+49.70 ± 7.94	0.10 ± 0.06	0.25 ± 0.05	221 ± 15 (100)	199 ± 73 (100)
**11**	593	7.6	+62.80 ± 0.78	0.37 ± 0.03	0.33 ± 0.03	145 ± 7 (91) 51 ± 5 (9)	143 ± 43 (96) 41 ± 9 (4)

^a^ Exact mass of compounds determined by mass spectrometry (MS): electrospray ionization positive ion mode (+ESI) *m*/*z* (^79^Br) ([M–Br]^+^ [22,26]. ^b^ zeta-potential determined for freshly prepared samples. ^c^ First set: for freshly prepared samples. ^d^ Second set: Two weeks after the first set; samples stored at r.t.

## Supplementary Materials

The following are available online at https://www.mdpi.com/1999-4923/11/3/115/s1, Table S1: Toxicity test data of tested 4-(*N*-alkylpyridinium)-1,4-DHPs **3**, **6**, **7**, and **9**–**11** on microorganism species; Figure S1: Representative example of determination of critical aggregation concentration (CAC) by DLS technique for 4-(*N*-hexadecylpyridinium)-1,4-DHP derivative **11**.
