# Supplementary material for: Contribution of Molecular Structure to Self-Assembling and Biological Properties of Bifunctional Lipid-Like 4-(N-Alkylpyridinium)-1,4-Dihydropyridines
Source: Pharmaceutics. 2019 Mar 12;11(3):115. doi: 10.3390/pharmaceutics11030115 (PMC6470589; doi:10.3390/pharmaceutics11030115)
Supplement: Supplementary file 1 [file pharmaceutics-11-00115-s001.pdf]

pharmaceutics

Supplementary Materials: Contribution of Molecular Structure to Self-Assembling and Biological Properties of Bifunctional Lipid-Like 4-(*N*-Alkylpyridinium)-1,4-Dihydropyridines

Martins Rucins, Pavels Dimitrijevs, Klavs Pajuste, Oksana Petrichenko, Ludmila Jackevica, Anita Gulbe, Signe Kibilda, Krisjanis Smits, Mara Plotniece, Dace Tirzite, Karlis Pajuste, Arkadij Sobolev, Janis Liepins, Ilona Domracheva and Aiva Plotniece *

Table 1. Toxicity test data of tested 4-(*N*-alkylpyridinium)-1,4-dihydropyridine derivatives **3**, **6**, **7**, and **9–11** on microorganism species; each compound boundary concentration is depicted in mM. Values of IC_{50} on HT-1080 and MH-22A cell lines are included for comparison and are not included in PCA analyses.

**Compound**		**3**	**6**	**7**	**9**	**10**	**11**
**Number of Propargyl Groups**		**0**	**1**	**2**	**2**	**0**	**1**
**Alkyl Chain Length in Pyridinium**		**12**	**12**	**2**	**12**	**16**	**16**
Compounds / Microorganisms		Boundary concentration, mM					
P. mirabilis	*gram negative*	0.001	1	0.1	0.01	0.01	0.0001
M. luteus	*gram positive*	0.001	0.0001	0.001	0.0001	0.001	0.001
P. aeruginosa	*gram negative*	0.01	0.01	0.01	0.01	10	0.01
B. subtilis	*gram positive*	0.01	0.0001	0.1	0.01	0.0001	0.0001
K. pneumoniae	*gram negative*	0.1	0.1	10	0.1	0.01	10
E. coli	*gram negative*	0.01	0.001	1	0.0001	0.01	0.01
S. cerevisiae	-	0.001	0.001	1	0.0001	0.0001	0.0001
Cell lines		Cytotoxicity, IC_{50}, µM					
HT-1080	-	0.037	0.004	1	0.002	-	-
MH-22A	-	0.015	0.001	1	0.001	-	-

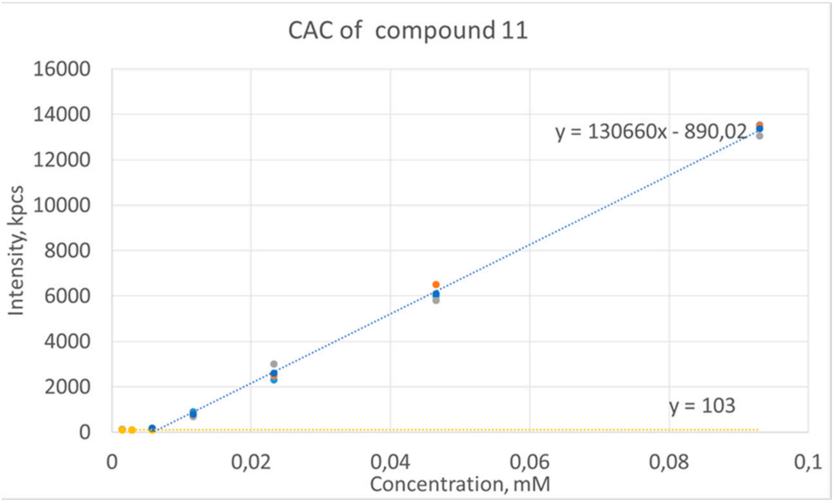

Figure 1. Representative example of determination of critical aggregation concentration (CAC) by DLS method for 4-(*N*-hexadecylpyridinium)-1,4-DHP derivative **11**.

Data of the critical micelle concentration (CAC) was used for data generation of Table 2.
